# Osimertinib combined with bevacizumab as the first‐line treatment in non‐small cell lung cancer patients with brain metastasis harboring epidermal growth factor receptor mutations

**DOI:** 10.1111/1759-7714.14880

**Published:** 2023-04-05

**Authors:** Ling Zhang, Yunhong You, Xueli Liu, Fengjuan Liu, Keke Nie, Youxin Ji

**Affiliations:** ^1^ Department of Oncology Affiliated Qingdao Central Hospital of Qingdao University, Qingdao Cancer Hospital Qingdao 266042 China

**Keywords:** brain metastasis, epidermal growth factor receptor tyrosine kinase inhibitor, non‐small cell lung cancer, osimertinib, target therapy

## Abstract

**Background:**

The efficacy and safety of osimertinib combined with bevacizumab in non‐small cell lung cancer (NSCLC) patients with brain metastasis harboring epidermal growth factor receptor (*EGFR*) mutations have not been fully studied.

**Methods:**

Treatment‐naïve NSCLC patients with brain metastasis harboring *EGFR*‐activating mutations were treated with osimertinib 80 mg oral daily and bevacizumab 15 mg/kg intravenously on day 1, repeated every 21 days, until disease progression, intolerable toxicity, or death. The primary endpoint was the median progression‐free survival (mPFS), and the secondary endpoints were the median overall survival (mOS), response rates, and toxicities. This study has been registered in ClinicalTrials.gov (NCT05104281) and is ongoing.

**Results:**

A total of 52 Chinese patients were enrolled, of whom 17 harbored *EGFR* 19 del and 35 harbored *EGFR* L858R mutation. The objective response rate (ORR) was 75.0% and the disease control rate (DCR) was 96.2%; the mPFS was 17.0 months (95% CI: 11.46–22.54), while the mOS was not reached. The mPFS was 20.0 months (95% CI: 14.56–25.44) and was 17.0 months (95% CI: 13.28–20.72) for patients harboring *EGFR* 19 del and *EGFR* L858R mutation (*p* = 0.844), respectively. The intracranial ORR was 82.7%, and the intracranial mPFS was 22.0 months (95% CI: 2.92–41.08).The main adverse events were mild‐to‐moderate hand‐foot syndrome, diarrhea, hypertension, and proteinuria. Three patients developed grade III proteinuria, while five patients developed grade III hypertension; they permanently discontinued bevacizumab treatment.

**Conclusions:**

Osimertinib combined with bevacizumab shows promising results in *EGFR*‐mutated NSCLC patients with brain metastasis, and the side effects are tolerable.

## Graphical abstract

Treatment‐naïve NSCLC patients with brain metastasis harboring *EGFR*‐activating mutations were treated with osimertinib and bevacizumab. A total of 52 Chinese patients were enrolled, of whom 17 harbored *EGFR* 19 del and 35 harbored *EGFR* L858R mutation. The objective response rate (ORR) was 75.0% and the disease control rate (DCR) was 96.2%; the mPFS was 17.0 months (95% CI: 11.46–22.54), while the mOS was not reached. The mPFS was 20.0 months (95% CI: 14.56–25.44) and was 17.0 months (95% CI: 13.28–20.72) for patients harboring *EGFR* 19 del and *EGFR* L858R mutation (*p* = 0.844), respectively. The intracranial ORR was 82.7%, and the intracranial mPFS was 22.0 months (95% CI: 2.92–41.08). Osimertinib combined with bevacizumab shows promising results in *EGFR*‐mutated NSCLC patients with brain metastasis, and the side effects are tolerable.

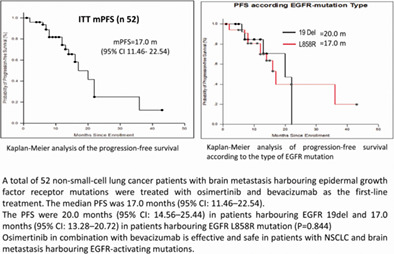

## INTRODUCTION

Lung cancer is the most common cancer type and the leading cause of cancer‐related deaths globally; non‐small cell lung cancer (NSCLC) accounts for 85%–90% of lung cancer cases.^1^, ^2^ The epidermal growth factor receptor (EGFR) gene somatic mutations in the kinase domain are the most common targetable gene of NSCLC, which are found in ~40%–58% and 17%–21% of Asian and Caucasian patients, respectively.^3^, ^4^, ^5^ Although EGFR tyrosine kinase inhibitors (EGFR‐TKIs) have become the recommended treatment strategy for patients with NSCLC harboring *EGFR* mutations, resistance would eventually occur with progression‐free survival (PFS) durations of 9.5–13.1 months for those treated with first‐ or second‐generation EGFR‐TKIs and 18.9 months for those treated with osimertinib.^6^, ^7^, ^8^, ^9^ To prolong the PFS and overall survival (OS) of locally advanced or metastatic NSCLC with *EGFR* somatic mutations, laborious effects have been made continuously in combined models in recent years.^10^, ^11^, ^12^ Since further improvements in treatment outcomes are required, the concomitant use of EGFR‐TKIs and antiangiogenesis agents has been considered. Gefitinib or erlotinib combined with bevacizumab prolonged the PFS to 16.0 months in advanced‐stage NSCLC patients with *EGF*R‐activating mutations, but the OS was not significantly different.^10^, ^13^

Osimertinib, a third‐generation EGFR‐TKI, demonstrated superior PFS and OS compared with erlotinib or gefitinib as the initial treatment for patients with *EGFR*‐mutant lung cancers.^8^ Previous phase I/II studies on first‐line or second‐line therapy with osimertinib and bevacizumab did not show prolonged PFS in NSCLC patients with *EGFR* or *EGFR* T790M mutations.^12^, ^14^ These results were not confirmed by a large phase III, randomized study, and growing evidence suggests that patients with *EGFR* gene mutation‐positive NSCLC are prone to developing brain metastases, with the frequency ranging from 44% to 63%.^15^, ^16^ Brain metastasis is a poor prognostic predictor of lung cancer,^8^, ^17^ and the efficiency of first‐generation EGFR‐TKIs combined with bevacizumab for *EGFR* mutation patients with brain metastasis remains controversial;^13^, ^18^ therefore, this study aimed to determine the efficacy and toxicities of osimertinib plus bevacizumab in patients with NSCLC with brain metastasis harboring *EGFR*‐activating mutations.

## METHODS

### Study design and patient selection

This is an ongoing, two‐center study. Patients enrolled in the study were histologically confirmed as having metastatic nonsquamous NSCLC with brain metastasis and were treatment naïve, with *EGFR* exon 19 del or L858R mutations confirmed via next‐generation sequencing (NGS) of biopsied tissues and/or blood. Computed tomography (CT)‐guided core needle biopsy was performed, DNA was extracted from 15 × 5 μm sliced sections of formalin‐fixed, paraffin‐embedded tumor tissue, and the tumor area was evaluated by a pathologist. For adequate sequences and mutation detection, at least 20% of the tumor area on each slice was set as the minimum. A total of 10 mL of blood were drawn and centrifuged for sequencing control and germline gene mutation tests. NGS was performed using HiSeq3000/HiSeq4000 Illumina techniques. Approximately 4278 exons of 288 common genes; intron, promoter, and fusion of 38 genes; coding area of 728 genes were tested for somatic mutations. A total of 11 germline mutations were identified. The ultra‐deep coverages of genes of interest were 1000× for tumor tissue and 10 000× for serum.

Participants were required to have at least one lesion in the lung and brain, which could accurately be measured in at least one dimension (longest diameter to be recorded) of ≥10 mm with spiral CT or magnetic resonance imaging (MRI). Patients with oligometastases in the brain and regional lung disease, which were suitable for surgical resection, were excluded. Meanwhile, participants aged ≥18 years, who had adequate organ and bone marrow function and had an Eastern Cooperative Oncology Group (ECOG) performance status score ≤2, were included in the study.

Bevacizumab (Avastin, Roche) 15 mg/kg was administered intravenously on day 1 and repeated every 21 days; osimertinib (Tagrisso, AstraZeneca) 80 mg was administered orally once a day. Treatment was continued until disease progression, intolerable toxicity, or death occurred. Reduction of bevacizumab dose to 7.5 mg/kg or osimertinib dose to 80 mg every other day was permitted.

Brain irradiation with whole‐brain radiotherapy (WBRT) or stereotactic radiosurgery (SRS) was indicated when patients had neurological symptoms or when it was required by a physician. The primary endpoint was PFS, and the secondary endpoints were OS, response rate, and toxicities.

### Outcomes and assessment

The primary endpoint of the study was PFS, while the secondary endpoints were OS, response rate, and toxicities. Responders were defined as those who had complete or partial response. PFS was measured from the first day of treatment until the first objective sign of disease progression or patient death, while OS was measured from the day of treatment to the day of patient death. Tumor responses, which were evaluated based on the Response Evaluation Criteria in Solid Tumor version 1.1 (RECIST version 1.1),^19^ were observed during the trial period and classified as follows: complete response (disappearance of tumor lesions), partial response (a decrease of at least 30% in the sum of tumor lesion sizes), stable disease (steady state of disease), or progressive disease (an increase of ≥20% in the sum of tumor lesion sizes). All adverse events were recorded and classified by grade according to the National Cancer Institute Common Terminology Criteria for Adverse Events, version 5.0.^19^

Tumor measurements using chest CT or MRI were performed at baseline and every 8–12 weeks thereafter. Patient compliance, treatment safety, and side effects were assessed every 2–3 weeks, every check‐up.

### Statistical analysis

Based on the Cox proportional hazards model and taking into account the influence of sex (male or female), Eastern Cooperative Oncology Group (ECOG) performance status score, hazard ratio, and 95% confidence interval (CI) were calculated in the full analysis population. The PFS and OS curves were analyzed using SigmaPlot 14.0 (Systat Software Inc.). The Kaplan–Meier log‐rank test was used in the intention‐to‐treat (ITT) population. The Kaplan–Meier method was used for survival analyses, and the log‐rank test was used to test for significance. The response rate and treatment‐related adverse events were assessed using the Fisher's exact test. All enrolled patients who received at least one dose of the study treatment were included in the safety analysis. Periodic safety monitoring and interim efficacy assessments were performed by an independent data monitoring committee. This trial has been registered in ClinicalTrials.gov (NCT05104281) and is ongoing.

The protocol and all modifications were approved by the Ethics Committee of the Affiliated Qingdao Central Hospital of Qingdao University on October 18, 2018 (approval number: KY202206102) and were performed in compliance with the provisions of Good Clinical Practice guidelines, the Declaration of Helsinki, and local laws. Informed consent was obtained from all patients prior to enrolment.

## RESULTS

A total of 71 patients were screened and 52 patients were subsequently enrolled in the study, of whom 38.5% (20/52) were male, and 61.5% (32/52) were female. The median age was 64.5 years, and 51.9% (27/52) of the patients were aged <65 years old. Approximately 90.4% (47/52) of the patients had adenocarcinomas, and 65.4% had an ECOG performance status score of 2. *EGFR* 19 del and L858R mutations were observed in 32.7% and 67.3% of the patients, respectively. All patients were newly diagnosed and treatment naïve. The demographic characteristics of patients are presented in Table 1.

**TABLE 1:** Clinicopathological features and patient factors.

Factors	No. of patients (*n* = 52) (%)
Gender	
Male	20 (38.5)
Female	32 (61.5)
Age (year)	
<65	27 (51.9)
≥65	25 (48.1)
ECOG performance status	
0	2 (3.8)
1	16 (30.8)
2	34 (65.4)
Histology subtype	
Adenocarcinoma	47 (90.4)
Large cell	4 (7.7)
NOS	1 (1.9)
*EGFR* mutation	
19 del	16 (30.8)
19 del + T790M	1 (1.9)
L858R	32 (61.5)
L858R + T790M	2 (3.8)
L858R + L861Q	1 (1.9)
Number of brain metastasis	
1	11 (21.2)
2–3	14 (26.9)
4–5	18 (34.6)
>5	9 (17.3)

Abbreviations: ECOG, Eastern Cooperative Oncology Group; EGFR, epidermal growth factor receptor; NOS, not otherwise specified.

**FIGURE 1:**
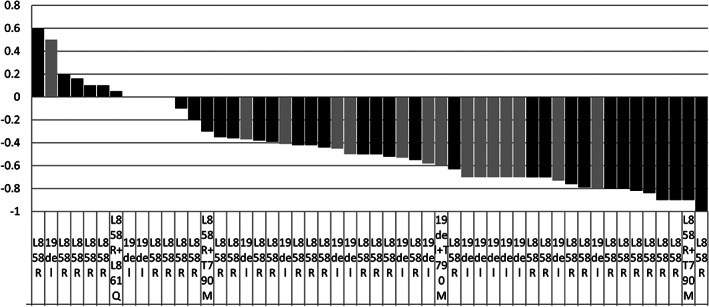
Changes in the target tumor.

**FIGURE 2:**
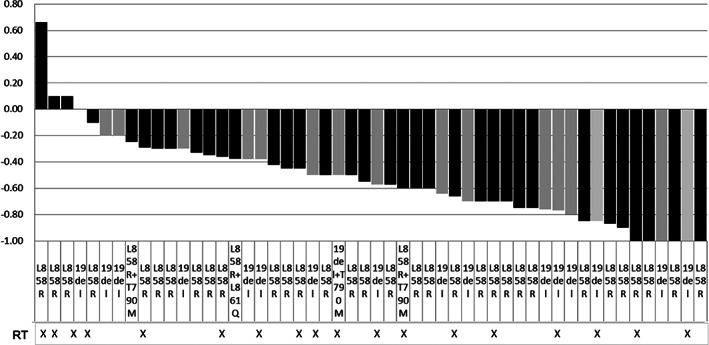
Changes in the intracranial disease.

The objective response rate (ORR) (complete or partial response) was 76.9% (40/52), which was assessed by the study investigators according to RECIST version 1.1, with a stable disease rate of 19.2% (10/52) and a disease control rate (DCR) of 96.2% (50/52). The median depth‐of‐response rate of the tumor (the nadir of tumor response) was 50.0% (Figure 1). The intracranial response rate (IC‐ORR) was 82.7% (43/52), while the intracranial DCR was 98.1% (51/52) (Table 2). Approximately 34.6% (18/52) of the patient underwent brain irradiation, of whom 38.9% (7/18) underwent SRS, while 61.1% (11/18) received WBRT. The intracranial PFS (IC‐PFS) was 22.0 months, and the median depth‐of‐response rate of intracranial lesions was 56.0% (Figure 2).

**TABLE 2:** Summary of efficiency measures.

Systemic/intracranial respons	19 del +/− T790M (*n* = 17)	L858R +/− T790M/L861Q (*n* = 35)	Total (*n* = 52)
Systemic tumor response (%)			
CR	0 (0)	2.9 (1/35)	1.9 (1/52)
PR	88.2 (15/17)	68.6 (24/35)	75.0 (39/52)
SD	11.8 (2/17)	22.9 (8/35)	19.2 (10/52)
PD	0 (0)	5.7 (2/35)	3.8 (2/52)
DCR	100 (17/17)	94.3 (33/35)	96.2 (50/52)
Intracranial response (%)			
iCR	11.8 (2/17)	11.4 (4/35)	11.5 (6/52)
iPR	70.6 (12/17)	71.4 (25/35)	71.2 (37/52)
iSD	17.6 (3/17)	14.3 (5/35)	15.4 (8/52)
iPD	0	2.9 (1/35)	1.9 (1/52)
iDCR	100 (17/17)	97.1 (34/35)	98.1 (51/52)
Median osimertinib oral days	341	242	292
Median bevacizumab cycles	11	12	11.5

Abbreviations: CR, complete response; DCR, disease controlrate; iCR, intracranial CR; iPR, intracranial PR; iSD, intracranial SD; iPD, intracranial PD; iDCR, intracranilal DCR; PD, progressive disease; PR, partial response; SD, stable disease.

**FIGURE 3:**
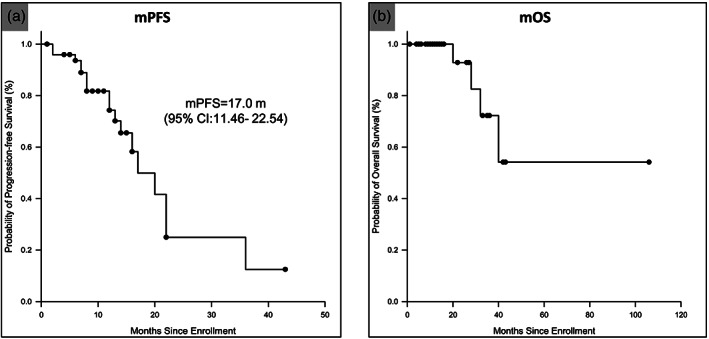
Kaplan–Meier analysis of the (a) progression‐free survival (PFS) and (b) overall survival (OS) in the full analysis set.

In 52 enrolled patients, the median PFS was 17.0 months (95% CI: 11.46–22.54) (Figure 3a), the median OS was not reached, and four events occurred at the last follow‐up on February 11, 2023 (Figure 3b).

**FIGURE 4:**
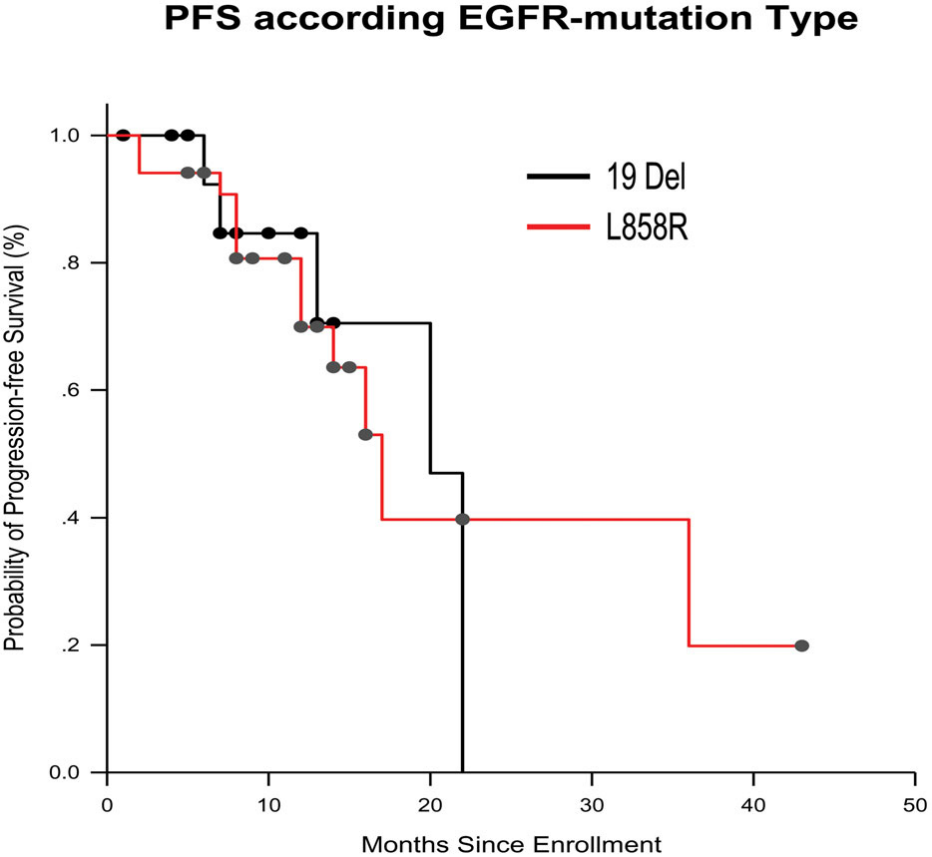
Kaplan–Meier analysis of progression‐free survival (PFS) according to the type of epidermal growth factor receptor (EGFR) mutation.

In the subgroup analysis, the PFS times were 20.0 months (95% CI: 14.56–25.44) in patients harboring *EGFR* 19 del and 17.0 months (95% CI: 13.28–20.72) in patients harboring *EGFR* L858R mutation (*p* = 0.844) (Figure 4); meanwhile, OS was not reached in both groups.

The side effects were mild‐to‐moderate hand‐foot syndrome, diarrhea, hypertension, and proteinuria. Three patients developed grade 3 proteinuria, while five patients developed grade 3 hypertension, for which bevacizumab treatment was permanently discontinued. Hand‐foot syndrome occurred in 80.8% (42/52) of the patients, which was the most common adverse effect, but most patients had grade I or II adverse effects. Five patients acquired paronychia, all of whom showed improvement after dose reduction and lotion embrocation (Table 3).

**TABLE 3:** Summary of adverse events.

Adverse events case (%) (*N* = 52)		
	All grade	Grade 3–4
Nausea	5 (9.6)	0 (0)
Loss of appetite	9 (17.3)	0 (0)
Stomatitis	6 (11.5)	0 (0)
Diarrhea	4 (7.7)	0 (0)
Paronychia	5 (9.6)	0 (0)
Hypertension	12 (23.1)	5 (9.6)
Hand‐foot syndrome	41 (78.8)	1 (1.9)
Gingiva bleeding	7 (13.5)	0 (0)
Proteinuria	11 (21.2)	3 (5.8)
Headache	7 (13.5)	0 (0)
Faintness	37 (71.2)	0 (0)

## DISCUSSION

*EGFR* mutations are considered the most robust predictive biomarkers for clinical and radiographic responses to EGFR‐TKIs in clinical practice.^7^, ^8^, ^9^ Bevacizumab is a vascular endothelial growth factor A (VEGFA) monoclonal antibody that combines with VEGFA, attenuates VEGFA‐dependent tumor blood vessel formation, normalizes tumor blood vessels, prompts tumor cell apoptosis, inhibits tumor angiogenesis, and finally shrinks the tumor.^20^, ^21^ It changes the tumor vessel physiology, thus increasing the intratumoral uptake of drugs. Previous studies of the administration of bevacizumab have demonstrated a clinical benefit for both primary brain tumor and brain metastasis in patients with advanced NSCLC, probably as a result of suppression of tumor angiogenesis and reduction of intracranial vasogenic edema.^22^, ^23^ In several studies, the addition of bevacizumab to first‐generation EGFR‐TKIs (gefitinib or erlotinib) significantly prolonged the PFS in patients with NSCLC with *EGFR*‐activating mutations compared with gefitinib or erlotinib alone; the OS was not significantly changed.^10^, ^13^ Previous studies showed that the ORR or PFS of the osimertinib plus bevacizumab group was not superior to that of the osimertinib alone group in treatment‐naïve patients harboring *EGFR*‐activating mutations, or in patients resistant to gefitinib or erlotinib who harbored the *EGFR*‐T790M mutation.^12^, ^14^ Brain metastases develop in up to 30% of patients with advanced‐stage NSCLC.^16^, ^24^, ^25^ Although the brain is one of the most frequent metastatic sites of lung cancer, only a few treatment options are available.^26^ Up to 40% of NSCLC patients with *EGFR* mutations treated with EGFR‐TKIs showed disease progression in the central nervous system, such as brain metastases or leptomeningeal metastases.^27^ Patients with brain metastasis harboring EGFR T790M, in whom osimertinib was used alone, had reasonable IC‐ORR and IC‐DCR, which reached 55.0% and 77.5%, respectively; however, the IC‐PFS was only 7.6 months.^27^ Dutta et al.^28^ reported that EGFR‐TKIs alone or EGFR‐TKIs with irradiation had a similar ORR and intracranial PFS (IC‐PFS); meanwhile, patients with high intracranial burden and neurological symptoms at diagnosis had similar IC‐PFS and OS compared with those with low burden and absence of neurological symptoms. In our study, the IC‐ORR was 82.7%, while the IC‐PFS reached 22.0 months, which was comparable to the extracranial ORR and extracranial PFS. All patients were treated with osimertinib and bevacizumab, and 34.6% (18/52) received brain external beam irradiation when neurological symptoms developed during the treatment course, which might have contributed to the higher IC‐ORR and longer IC‐PFS in this group.

To the best of our knowledge, this study is the first to report the efficacy of osimertinib in combination with bevacizumab as the first‐line treatment in NSCLC patients with brain metastasis harboring *EGFR* mutations. It had a high response rate and durable extracranial PFS and IC‐PFS. However, the present study had some limitations. This was a one‐arm study with fewer participants, which can potentially cause selection bias. Compared with other studies, the present study had a relatively short follow‐up period, which can affect the statistical power. The predictive value of osimertinib plus bevacizumab in the treatment of patients with NSCLC with brain metastasis harboring *EGFR* mutations should be further explored in a randomized setting.

In conclusion, osimertinib in combination with bevacizumab is effective and safe in patients with NSCLC and brain metastasis harboring *EGFR*‐activating mutations. However, randomized studies with more patients are required to confirm this finding.

## AUTHOR CONTRIBUTIONS

Keke Nie and Youxin Ji designed the study. Ling Zhang and Yunhong You collected the clinical data. Xueli Liu and Fengjun Liu performed the statistical study and preparation of figures. Keke Nie and Youxin Ji drafted this manuscript. All authors read and approved the final manuscript.

## CONFLICT OF INTEREST STATEMENT

The authors declare no conflicts of interest.

## ETHICS STATEMENT

This study was approved by the Ethics Committee of the Affiliated Qingdao Central Hospital of Qingdao University and was performed in compliance with the provisions of the Good Clinical Practice guidelines, the Declaration of Helsinki, and local laws.

## Data Availability

The datasets generated and/or analyzed during the current study are available from the corresponding author upon reasonable request.
